# Homoharringtonine, a clinically approved anti-leukemia drug, sensitizes tumor cells for TRAIL-induced necroptosis

**DOI:** 10.1186/s12964-015-0103-0

**Published:** 2015-04-29

**Authors:** Stephan Philipp, Justyna Sosna, Johannes Plenge, Holger Kalthoff, Dieter Adam

**Affiliations:** Institut für Immunologie, Christian-Albrechts-Universität, Kiel, Germany; Institut für Experimentelle Tumorforschung, Christian-Albrechts-Universität, Kiel, Germany

**Keywords:** Homoharringtonine, Necroptosis, TRAIL, Regulated necrosis

## Abstract

**Background:**

One hallmark of cancer cells is their ability to evade physiologic signals causing regulated cell death (RCD). Correspondingly, TRAIL-based therapies to eliminate human cancer cells via enforced induction of apoptosis have been established and represent a promising approach in anti-cancer research. However, due to frequently appearing intrinsic or acquired resistances of tumor cells against apoptosis, TRAIL-based apoptotic strategies for the treatment of cancer patients have shown limited efficacy. As a potential alternative, regulated necrosis (and necroptosis triggered e.g. by TRAIL receptors 1/2) has recently gained considerable attention. Regulated necrosis represents a mode of RCD molecularly distinct from apoptosis whose potential in anti-cancer therapy is almost uncharacterized. Since in most cancer cells survival pathways counteract the effects of TRAIL-induced RCD, sensitizers such as cycloheximide (CHX) are frequently added in cell culture to overcome this problem. Unfortunately, those sensitizers are cytotoxic and therefore not suitable for the treatment of cancer patients. Here, we have alternatively employed homoharringtonine (HHT), a plant alkaloid which was recently approved by the U. S. Food and Drug Administration to treat patients with chronic myeloid lymphoma.

**Results:**

We show that HHT is an efficient sensitizer for TRAIL-induced necroptosis in multiple human cancer cell lines. In addition, HHT-enhanced TRAIL-mediated necroptosis occurs via the same signaling pathways (involving RIPK1/RIPK3/MLKL) as CHX-enhanced necroptosis. Importantly, consecutive treatment schedules of necroptosis and apoptosis in either combination revealed remarkable additive effects not reached by repetitive apoptotic treatments alone.

**Conclusions:**

Taken together, our data demonstrate that HHT can replace harmful substances such as CHX to sensitize human cancer cells to TRAIL-induced necroptosis. Thus, HHT represents a promising enhancer in TRAIL-based necroptotic anti-cancer therapies also in patients.

## Background

Regulated cell death (RCD) is a critical element in the manifestation of many human diseases, especially tumor formation. Apoptosis is the best described form of RCD and depends on the activation of caspases [1,2]. In contrast, necrosis has been classified for decades as a purely accidental and uncontrolled type of cell death. Since the discovery in 1988 that upon treatment with tumor necrosis factor (TNF), cells showed either features of apoptosis or a necrotic balloon-like morphology without nuclear disintegration, the concept that RCD can also occur via regulated necrosis has been established by an increasing number of studies ([3,4] reviewed in [5]). Also, we and others have obtained evidence that regulated necrosis may represent an alternative pathway for the treatment of cancers that are resistant to apoptosis [6,7]. Because treatment with TNF is associated with strong systemic toxic side effects, the cytokine TNF-related apoptosis inducing ligand (TRAIL) has evolved as the most promising alterative for cancer therapy (TRAIL induces apoptosis in tumor cells while leaving non-transformed cells mostly unaffected) [8-10]. Different pre-clinical as well as phase I/II clinical studies demonstrated no systemic toxicity of TRAIL to organs and tissues [11,12]. Unfortunately, many tumor cells are resistant against TRAIL-induced apoptosis either intrinsically and/or via various anti-apoptotic signals from the tumor microenvironment. The increased expression of anti-apoptotic proteins such as Bcl-XL, cFLIP, or XIAP and the reduced expression of pro-apoptotic molecules such as caspases and Bid are just some examples of such protective signals [13]. Since TRAIL-induced necroptosis relies on completely distinct molecular pathways, it may therefore represent an effective alternative method to selectively eliminate these cancer cells [14-16].

Many recent studies have focused on boosting the efficiency of TRAIL-induced apoptosis against tumor cells by combined treatment with additional substances [7,17-19] to overcome their resistances. For example, CHX is widely used to sensitize tumor cells against TNF/TRAIL-induced apoptosis, and we have previously also used CHX to sensitize tumor cells against TRAIL-induced necroptosis [7]. Unfortunately, in animal models, CHX treatment causes severe side effects such as liver damage [20]. Therefore, alternative non-toxic sensitizing substances without such severe side effects are needed for an effective TRAIL based therapy of tumor patients.

In this study, we investigated the natural alkaloid HHT (also known as omacetaxine mepesuccinate) as a potential non-toxic sensitizer for TRAIL-induced necroptosis. In traditional Chinese medicine, HHT was obtained from extracts from the bark of cephalotaxus species and given to patients and the initial clinical study was reported by the Cephalotaxus Research Coordinating Group in 1976 [21]. HHT was used in the early 1980s to directly treat chronic myeloid leukemia (CML) patients in China [22]. Since then, patients have been successfully treated with HHT in multiple clinical studies [23-26]. Finally, HHT was recently approved by the U. S. Food and Drug Administration for treating patients with chronic or accelerated phase chronic myeloid leukemia with resistance and/or intolerance to two or more tyrosine kinase inhibitors [27,28]. Here, we show that HHT represents an efficient sensitizer for TRAIL-induced necroptosis in a panel of different tumor cell lines. These findings implicate that HHT/TRAIL-induced necroptosis may also be successfully utilized in future therapy regimens in cancer patients.

## Results

### HHT sensitizes human tumor cell lines to TRAIL-induced necroptosis

**Figure 1:**
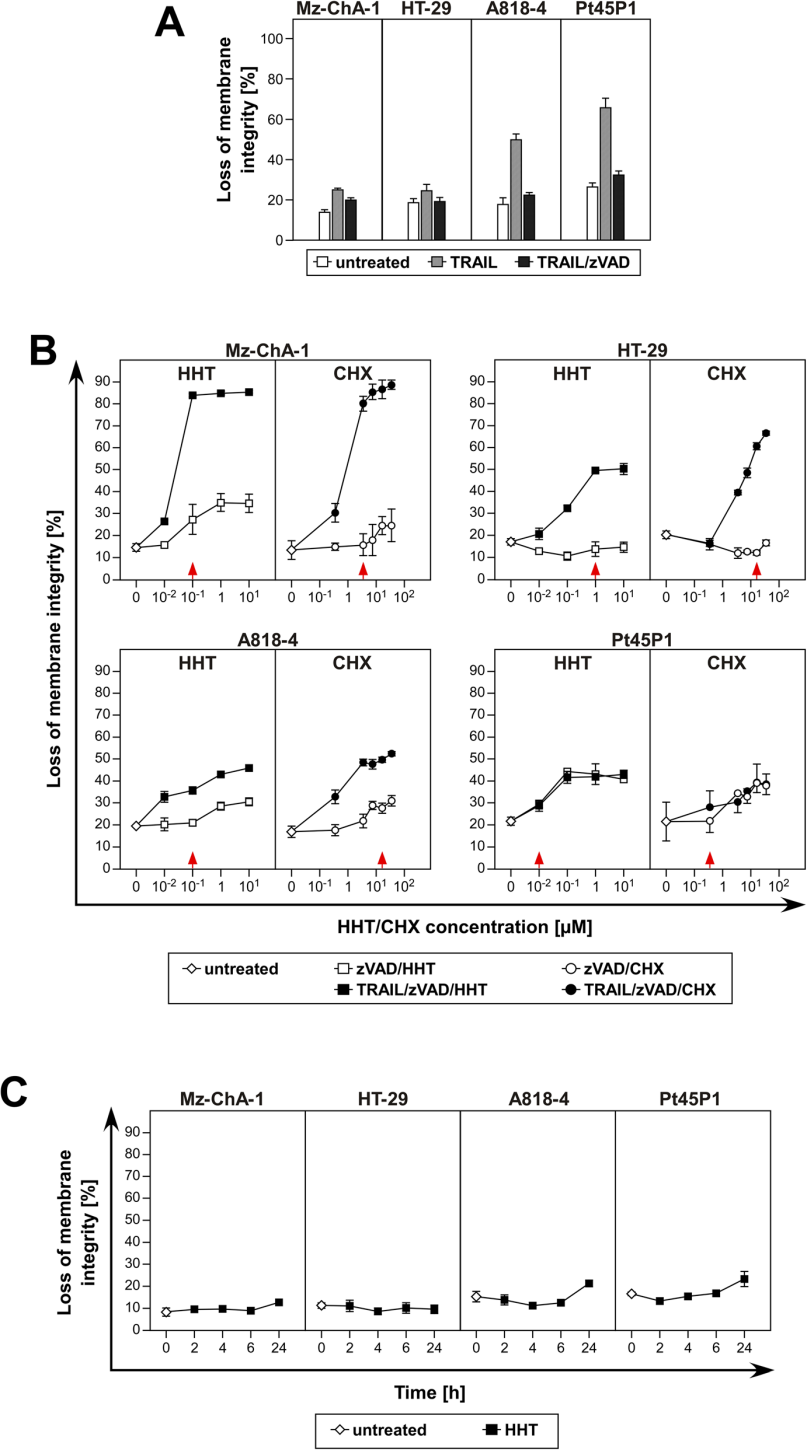
HHT sensitizes cancer cells for TRAIL-induced necroptosis. **(A)** Cells were treated with TRAIL with or without pretreatment of 50 μM zVAD-fmk to induce cell death in the absence of any additional sensitizers. **(B)** Cells were pretreated with 50 μM zVAD-fmk in combination with 0.01, 0.1, 1, and 10 μM of HHT or 0.36, 3.56, 7.12, 17.79 and 35.59 μM CHX (corresponding to 0.1, 1, 2, 5 and 10 μg/ml) for one hour before 100 ng/ml of TRAIL were added. **(C)** Cells were stimulated with optimized concentrations of HHT (red arrows in **(B)**) in a time course experiment to monitor the cytotoxic effects of HHT alone. Loss of membrane integrity (after 24 h for panels **A** and **B**, as indicated for panel **C)** was measured as a marker for cell death by flow cytometric detection of PI-positive cells. Each measurement represents the means of two independent experiments with three parallel determinations each, error bars indicate the corresponding standard deviations (SDs). Red arrows indicate concentrations used for further experiments.

We have previously demonstrated that the sensitivity of tumor cells for TRAIL-induced necroptosis can be enhanced by the protein biosynthesis inhibitor CHX [7]. Moreover, we and others have found that treatment with TRAIL normally activates caspase-dependent apoptosis. Since this would interfere with the analysis of regulated necrosis, caspases and apoptosis have to be actively inhibited, e.g. by addition of the broad-spectrum caspase inhibitor zVAD-fmk [7,14,29]. In a first set of experiments, we tested whether treatment of cells with zVAD-fmk in combination with TRAIL induced any cell death and compared this to the induction of apoptosis by treatment with TRAIL alone (Figure 1A). These experiments clearly demonstrate that treatment with zVAD/TRAIL is not sufficient to induce cell death in these human cancer cells and that sensitizers are indispensable. In the next step we investigated the sensitizing effect of increasing concentrations of HHT and CHX with or without addition of TRAIL and in the presence of zVAD-fmk (Figure 1B). The tumor cell lines tested in these experiments were initially selected due to their differential sensitivities to TRAIL-induced necroptosis they had shown in a previous study, ranging from very sensitive (Mz-ChA-1), well sensitive (HT-29), moderately sensitive (A818-4) to being resistant (Pt45P1) [7]. In the same study, the cell lines had also been verified for cell surface expression of TRAIL receptors 1 and 2. Consistent with this previous study, the four tumor cell lines proved highly (Mz-ChA-1), moderately (HT-29, A818-4) or not (Pt45P1) susceptible to TRAIL-induced necroptosis when sensitized either with HHT or with CHX. From the obtained data, we determined the optimal concentrations of HHT and CHX which induced the highest rate of TRAIL-mediated necroptosis while still being non-toxic in the absence of TRAIL as follows: Mz-ChA-1: HHT-0.1 μM, CHX-7.12 μM (corresponding to 2 μg/ml); HT-29: HHT-1 μM, CHX-17.79 μM (5 μg/ml); A818-4: HHT-0.1 μM, CHX-35.59 μM (10 μg/ml); Pt45P1: HHT-0.01 μM, CHX-0.36 μM (0.1 μg/ml)). These optimal sensitizing concentrations were subsequently used in the following experiments. To additionally clarify whether HHT had any effect on membrane integrity by itself, we monitored cell death in HHT-only treated cells over time (0, 2, 4, 6, 24 hours, using the optimized concentrations determined above). As shown in Figure 1C, HHT did not induce cell death by itself, except for a minimal increase in A818-4 and Pt45P1 cells at 24 h (the longest incubation time). Noteworthy, HHT is able to sensitize cells for TRAIL-induced necroptosis in the same manner as CHX, but in much lower concentrations (Mz-ChA-1: 71-fold, i.e., 0.1 μM HHT vs. 7.12 μM CHX, HT-29: 17-fold, i.e., 1 μM HHT vs. 17.79 μM CHX, A818-4: 350-fold, i.e., 0.1 μM HHT vs. 35.59 μM CHX, Pt45P1: 36-fold, i.e., 0.01 μM HHT vs. 0.36 μM CHX).

**Figure 2:**
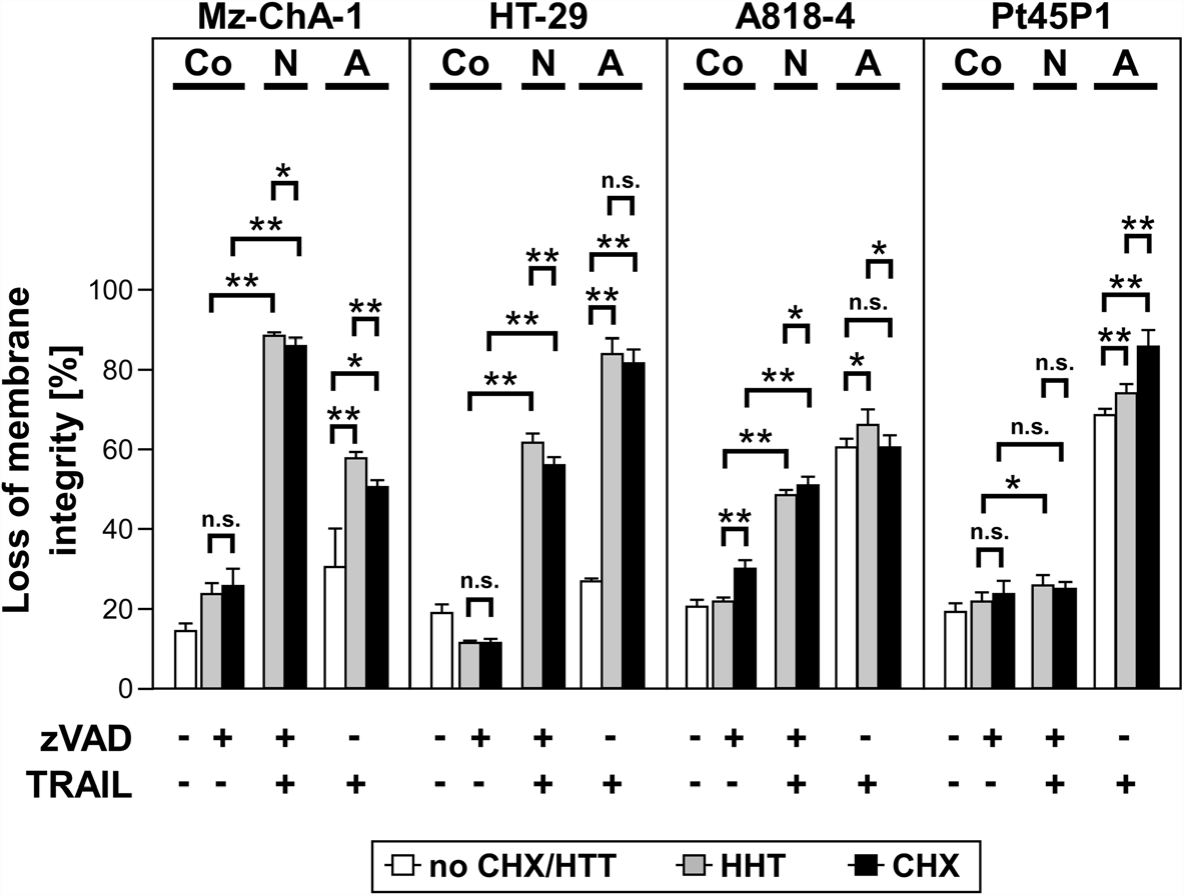
Comparison of the sensitizing effect of HHT or CHX on TRAIL-induced necroptosis vs. apoptosis in tumor cells. Cells were pretreated with 50 μM zVAD-fmk with or without HHT/CHX (Mz-ChA-1: HHT-0.1 μM, CHX-7.12 μM; HT-29: HHT-1 μM, CHX-17.79 μM; A818-4: HHT-0.1 μM, CHX-35.59 μM; Pt45P1: HHT-0.01 μM, CHX-0.36 μM) and after one hour, 100 ng/ml of TRAIL were added. After 24 h, loss of membrane integrity was measured as a marker for cell death by flow cytometric detection of PI-positive cells. Spontaneous cell death in untreated cells is shown for comparison. Each bar represent the means of two independent experiments with three parallel determinations each, error bars indicate the corresponding SDs. Asterisks indicate statistical significance (t-test; *, *P* < 0.05; **, *P* < 0.001; n.s., not significant). Co: Control, N: Necroptosis, A: Apoptosis.

We additionally validated these results in an independent experiment where we utilized the optimized concentrations of HHT and CHX to compare the sensitization for TRAIL-induced necroptosis with the ability of HHT and CHX to sensitize for TRAIL-induced apoptosis (Figure 2). As expected, all cell lines showed a comparable level of sensitization for necroptosis by the optimized concentrations of HHT and CHX. This was also observed for apoptosis (except for a slightly higher sensitization by CHX in Pt45P1 cells), underscoring the potential of HHT as a sensitizer also for apoptotic therapy regimens and in agreement with a previous study [30]. Notably, HHT and CHX were able to sensitize Mz-ChA-1 and HT-29 cells for both necroptosis and apoptosis, whereas A818-4 cells showed effective sensitization only for necroptosis, and Pt45P1 cells for neither mode of RCD (Figure 2). Therefore, sensitization by HHT or CHX for apoptosis does not automatically mean sensitization for necroptosis and vice versa.

**Figure 3:**
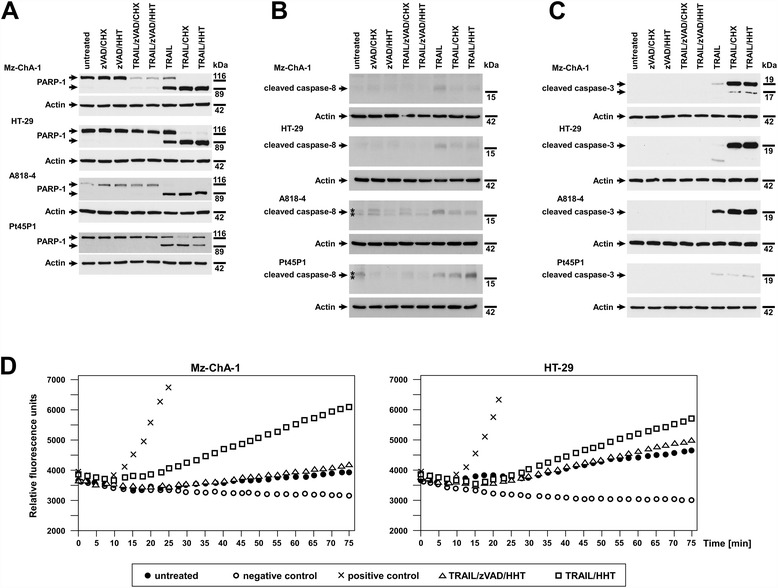
Treatment with TRAIL/zVAD/CHX or TRAIL/zVAD/HHT does not elicit apoptosis in tumor cells. **(A-C)** Cells were either left untreated or pretreated with 50 μM zVAD-fmk with or without HHT/CHX (same concentrations as given in Figure *2*). After one hour, 100 ng/ml of TRAIL were added as indicated. After 24 h of stimulation, complete cell lysates were prepared. Identical amounts of protein (20 μg) were loaded on each lane (from left to right: lane 1–3: negative controls, lane 4–5: necroptosis, lane 6–8: apoptosis as positive control) and PARP-1, cleaved caspase-8 and cleaved caspase-3 were detected in Western blots. **(A)** The antibody against PARP-1 recognizes the 116-kDa full-length protein and the cleaved 89-kDa form that is only visible in apoptotic lysates (TRAIL, TRAIL/CHX, TRAIL/HHT). **(B)** The antibody against caspase-8 recognizes the 18-kDa cleaved fragment that is present only in apoptotic lysates (TRAIL, TRAIL/CHX, TRAIL/HHT). Asterisks: non-specific double band that does not match to the size of the 18-kDa cleaved fragment of caspase-8. **(C)** The antibody against caspase-3 only recognizes the cleaved (active) form of caspase-3 that appears only in apoptotic lysates (TRAIL, TRAIL/CHX, TRAIL/HHT). Detection of actin served as a loading control. **(D)** Caspase-8 activity was measured in Mz-ChA-1 and HT-29 cells. Extracts were prepared from untreated (●) cells or from cells in which necroptosis (Δ) or apoptosis (□) had been induced as in **(A-C)**, except that pretreatment with zVAD-fmk was for 30 min and that stimulation time was 4 h. As a positive control (×), caspase activity in untreated extracts was stimulated by the addition of dATP and cytochrome c; samples containing only buffer but no extracts served as negative control (○). Caspase-8 activity was determined by measuring the cleavage of a fluorogenic peptide (zIETD-afc) in a time course over 75 minutes as described in Methods.

While performing the above experiments, the question arose whether RCD induced by TRAIL/zVAD/HHT or TRAIL/zVAD/CHX indeed occurred exclusively by necroptosis or whether the presence of HHT or CHX might still result in a (residual) apoptotic response, i.e., activation of caspases. We have previously demonstrated in several studies and in multiple cell systems that no features of apoptosis are detectable in the presence of 20 or 50 μM zVAD-fmk [4,14,31,32], but we nevertheless decided to additionally clarify this issue in the very cell lines utilized in this study. Poly(ADP-ribose) polymerase-1 (PARP-1) is a 116-kDa protein that is inactivated during apoptosis by caspase-3-mediated cleavage to an 89-kDa product [33]. Furthermore, the activation of caspase-8 as an initiator caspase as well as of caspase-3 as an executor caspase (i.e., cleavage from their precursors to the active fragments) represent critical steps in apoptosis. Therefore, the 89-kDa cleavage fragment of PARP-1 as well as cleaved fragments of caspase-8 and caspase-3 represent molecular markers for the occurrence of apoptosis. We induced necroptosis in Mz-ChA-1, HT-29, A818-4 and Pt45P1 cells by treating them with TRAIL/zVAD/CHX or TNF/zVAD/HHT. For control, we treated the cells with zVAD/CHX or zVAD/HHT alone, induced apoptosis by stimulation with TRAIL or with TRAIL/CHX, and additionally tested cells treated with TRAIL/HHT in Western blots for PARP-1 cleavage and for the presence of cleaved fragments of caspase-8 and caspase-3 (Figure 3A-C). In all cell lines, no increase in the levels of cleaved PARP-1 as well as cleaved caspase-8 and caspase-3 were detectable during TRAIL/zVAD/CHX- or TRAIL/zVAD/HHT-mediated RCD compared to the untreated and zVAD/CHX or zVAD/HHT-controls. In contrast, after induction of apoptosis by stimulation with TRAIL alone or in combination with CHX, cleavage products for PARP-1, caspase-8 and caspase-3 were clearly detectable (Figure 3A-C). As an additional parameter, we directly measured the activity of caspase-8 in Mz-ChA-1 and HT-29 cells (Figure 3D). Compared to untreated cells, caspase-8 activity remained more or less unchanged after stimulation with TRAIL/zVAD/HHT. In contrast, a clear increase in active caspase-8 was detectable in apoptotic samples treated with TRAIL/HHT. These results demonstrate that treatment with TRAIL/zVAD/CHX or TRAIL/zVAD/HHT indeed does not activate caspases in the tested cell lines. In conclusion, RCD in response to TRAIL/zVAD/CHX or TRAIL/zVAD/HHT occurs exclusively by necroptosis but not by apoptosis. As a further result of these experiments, treatment with TRAIL/HHT lead to the activation of caspase-8 and to a pronounced cleavage of PARP-1, caspase-8 and caspase-3 in all cell lines (Figure 3), demonstrating that HHT (like CHX) can also act as an enhancer of apoptotic RCD.

### HHT-enhanced TRAIL-induced necroptosis depends on activation of RIP kinases

**Figure 4:**
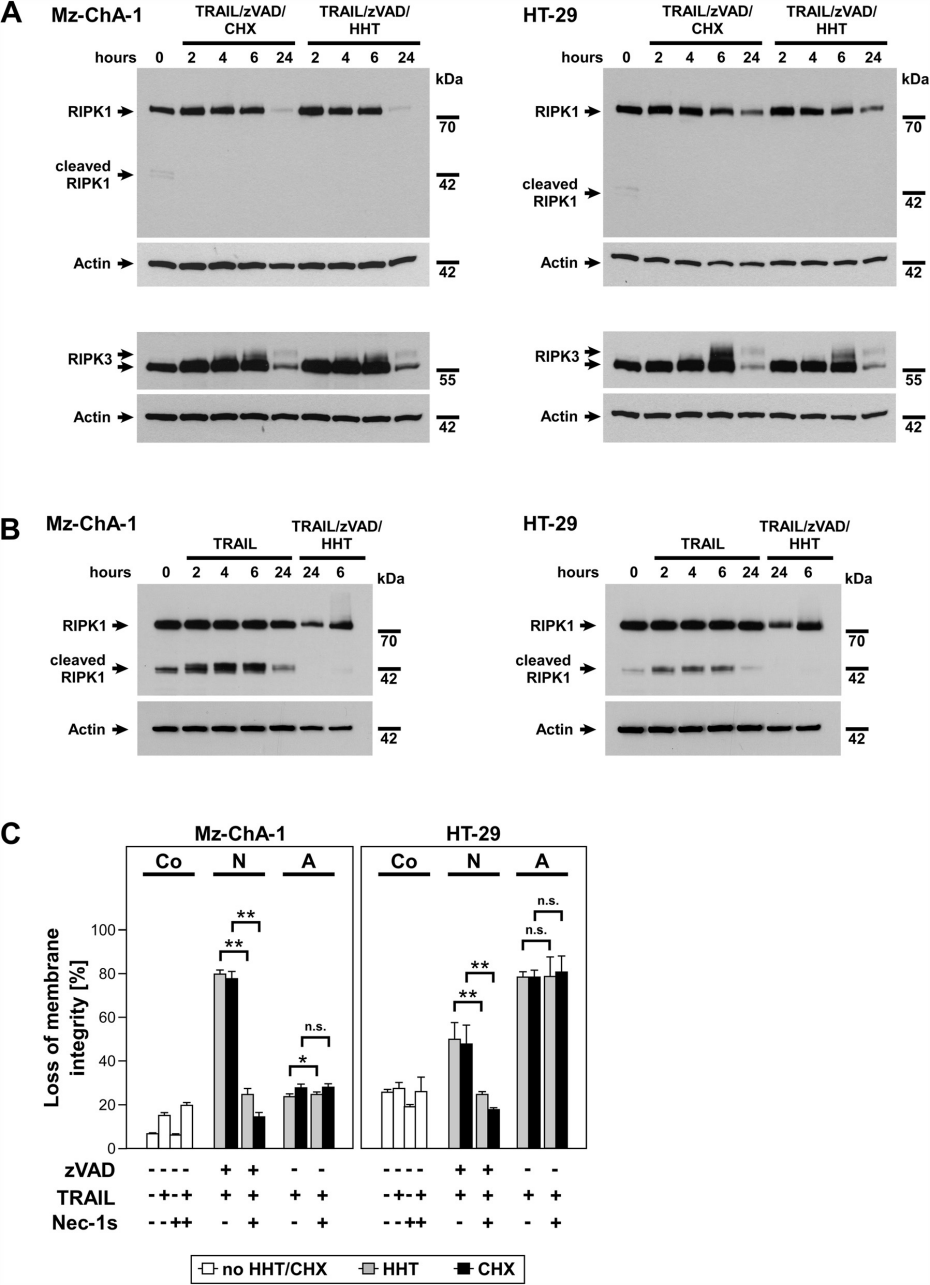
HHT-enhanced TRAIL-induced necroptosis depends on activation of RIPK1/RIPK3 and can be blocked by necrostatin-1s (Nec-1s). **(A, B)** Cells were pretreated with 50 μM zVAD-fmk with HHT (Mz-ChA-1: 0.1 μM; HT-29: 1 μM) or CHX (Mz-ChA-1: 7.12 μM; HT-29: 17.79 μM) or left untreated, and after one hour, 100 ng/ml of TRAIL were added. Cell lysates were prepared at the indicated time points after stimulation and RIPK1 (including its 42-kDa cleavage fragments) and RIPK3 were detected by Western blot. Detection of actin served as a loading control. **(C)** Cells were preincubated with 50 μM Nec-1s for one hour and stimulated as indicated in **(A)**. After 24 h, loss of membrane integrity was measured as a marker for cell death by flow cytometric detection of PI-positive cells. Cell death in the corresponding controls is shown for comparison (white tlsb-.1pt?>bars). Each bar represents the means of two independent experiments with three parallel determinations each, error bars indicate the corresponding SDs. The addition of Nec-1s significantly reduced the level of necroptosis compared to cells not treated with Nec-1s, regardless of the presence of HHT or CHX. Asterisks indicate statistical significance (t-test; *, *P* < 0.05; **, *P* < 0.001; n.s., not significant). Co: Control, N: Necroptosis, A: Apoptosis.

For the following experiments, we selected Mz-ChA-1 and HT-29 cells since these cells were most susceptible to necroptosis (see Figure 1). Activation of receptor interacting protein kinase 1 and 3 (RIPK1/RIPK3) is a crucial step in the signaling pathways of necroptosis [34]. We performed time kinetic experiments (0, 2, 4, 6 and 24 hours) to monitor whether the addition of HHT caused alteration in these signaling pathways, e.g., whether RIPK1 and RIPK3 activation did still occur as expected in HHT-sensitized cells undergoing necroptosis (Figure 4A). Within two hours after stimulation of Mz-ChA-1 or HT-29 cells with TRAIL/zVAD/CHX (included for control) or TRAIL/zVAD/HHT, higher molecular weight forms (smear), in particular for RIPK3, were observed in Western blots. As the most likely explanation, this increase in molecular weight is due to phosphorylation and activation of these proteins when they trigger necroptosis. The intensity of the detected RIPK1 and RIPK3 bands decreased after 24 hours of stimulation, indicating a termination of necroptotic signaling in the late to end stages of cellular disintegration (Figure 4A). Notably, inactivation of RIPK1 by cleavage to a fragment of 42 kDa has been reported during apoptosis [35]. Correspondingly, we observed a significant increase of the 42-kDa RIPK1 cleavage fragment (which was present at low levels already in untreated cells (Figure 4A, B)) when we induced apoptosis with TRAIL in Mz-ChA-1 and HT-29 cells. In contrast, no inactivation of RIPK1 by cleavage was detectable when necroptosis was triggered by TRAIL/zVAD/CHX or TRAIL/zVAD/HHT (Figure 4A, B), consistent with the importance of functional RIPK1 in necroptosis. To validate whether activation of RIP kinases is indeed essential also for progression of HHT-enhanced necroptosis, cells were treated with necrostatin-1s (Nec-1s) which is an efficient inhibitor of RIPK1 [36,37]. In our experiments, Nec-1s was able to completely block TRAIL-induced necroptosis (but had no effect on apoptosis; *P* for Mz-ChA-1 cells was 0.045 and thus only marginally below the significance threshold of 0.05) either sensitized by HHT or CHX (Figure 4C), clearly confirming the relevance of RIPK1 in HHT- or CHX-enhanced TRAIL-induced necroptosis.

### HHT-enhanced TRAIL-induced necroptosis occurs via phosphorylation and activation of MLKL

**Figure 5:**
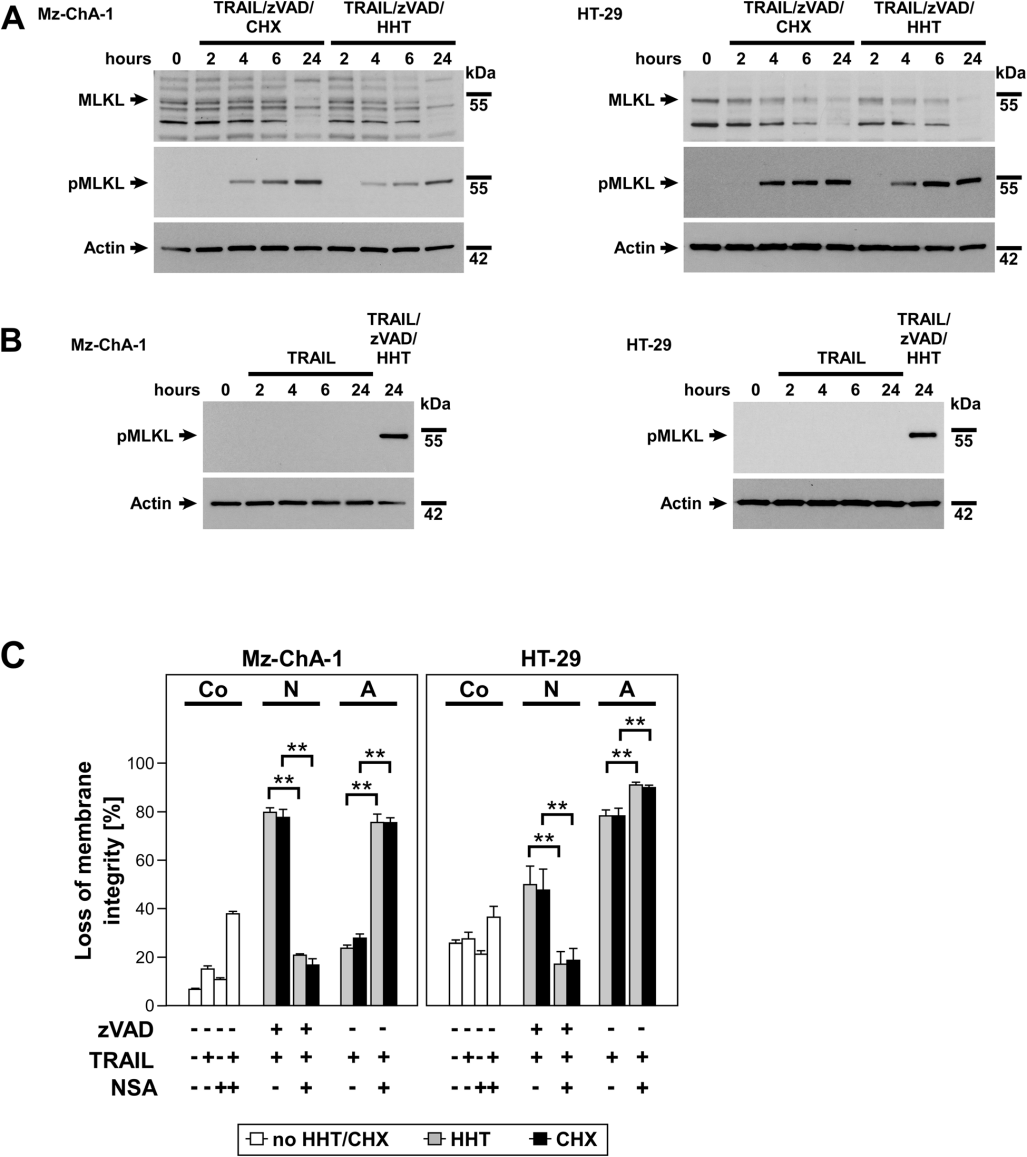
HHT-enhanced TRAIL-induced necroptosis occurs via activation of the downstream signaling component MLKL. **(A)** Activation of MLKL was checked by Western blot of its unphosphorylated and phosphorylated forms during a time course of 24 h. Cells were prestimulated with 50 μM zVAD-fmk with HHT (Mz-ChA-1: 0.1 μM; HT-29: 1 μM) or CHX (Mz-ChA-1: 7.12 μM; HT-29: 17.79 μM), followed by addition of 100 ng/ml of TRAIL after one hour. Cell lysates were prepared at the indicated time points after stimulation and loaded on a SDS gel (20 μg per lane). The unphosphorylated and phosphorylated forms of MLKL were detected by Western blot and detection of actin served as loading control. **(B)** Phosphorylation of MLKL was not detected in Mz-ChA-1 and HT-29 cells after induction of apoptosis with 100 ng/ml TRAIL for the indicated time points. 20 μg of protein was analyzed per lane, actin served as loading control and necroptotic samples were loaded for control. **(C)** Cells were preincubated with necrosulfonamide (NSA, specific inhibitor of MLKL) for one hour and afterwards stimulated as indicated in **(A)**. After 24 h, loss of membrane integrity was measured as a marker for cell death by flow cytometric detection of PI-positive cells. Cell death in the corresponding controls is shown for comparison (white bars). Each bar represents the means of two independent experiments with three parallel determinations each, error bars indicate the corresponding SDs. The addition of NSA significantly reduced the level of necroptosis compared to cells not treated with NSA, regardless of the presence of HHT or CHX. Asterisks indicate statistical significance (t-test; **, *P* < 0.001). As part of the same experiment shown in Figure 4C, the same negative control values were used. For clarity of presentation, the data of Nec-1s- (Figure 4C) and NSA-stimulated cells are shown in two separate figures. Co: Control, N: Necroptosis, A: Apoptosis.

In the next step we checked whether crucial components of necroptotic signaling downstream of RIPK1 and RIPK3 are modulated in HHT-enhanced necroptosis and compared this to CHX-enhanced necroptosis in Mz-ChA-1 and HT-29 cells. For this purpose, we investigated mixed lineage kinase domain-like protein (MLKL) which is a critical downstream target of RIPK3 [38,39]. Because it is well established that MLKL is phosphorylated during necroptosis leading to its activation, we used an antibody that exclusively recognizes the phosphorylated form of MLKL (pMLKL) and compared that to signals from an MLKL antibody that recognizes the unphosphorylated form of the protein [39,40]. In unstimulated lysates and lysates from cells two hours after stimulation either with TRAIL/zVAD/CHX (included for control) or TRAIL/zVAD/HHT, we were not able to detect pMLKL (Figure 5A). In contrast, after four hours of stimulation, pMLKL was detectable and the signal significantly increased over time, which clearly shows that MLKL gets activated by phosphorylation in HHT (as well as CHX)-enhanced TRAIL-induced necroptosis. Conversely, we were not able to detect any pMLKL in TRAIL-induced apoptotic samples (Figure 5B). We then used necrosulfonamide (NSA) that covalently binds to MLKL in human cells to block execution of regulated necrosis [39]. The data obtained in this experiment clearly showed that in both cell lines, NSA was able to completely block TRAIL-induced necroptosis sensitized with HHT or CHX (Figure 5C). Interestingly, NSA sensitized the cells for apoptosis in the control as well as when HHT or CHX were present. This may point to further yet unknown targets of NSA and/or to an additional undiscovered role of MLKL in the suppression of apoptosis. Taken together, we show that HHT-enhanced TRAIL-induced necroptosis leads to phosphorylation of MLKL as a known critical component of the necroptotic signaling pathway, comparable to CHX-enhanced TRAIL-induced necroptosis.

### Downregulation of RIPK3 and MLKL protects from TRAIL/zVAD/HHT-mediated necroptosis.

**Figure 6:**
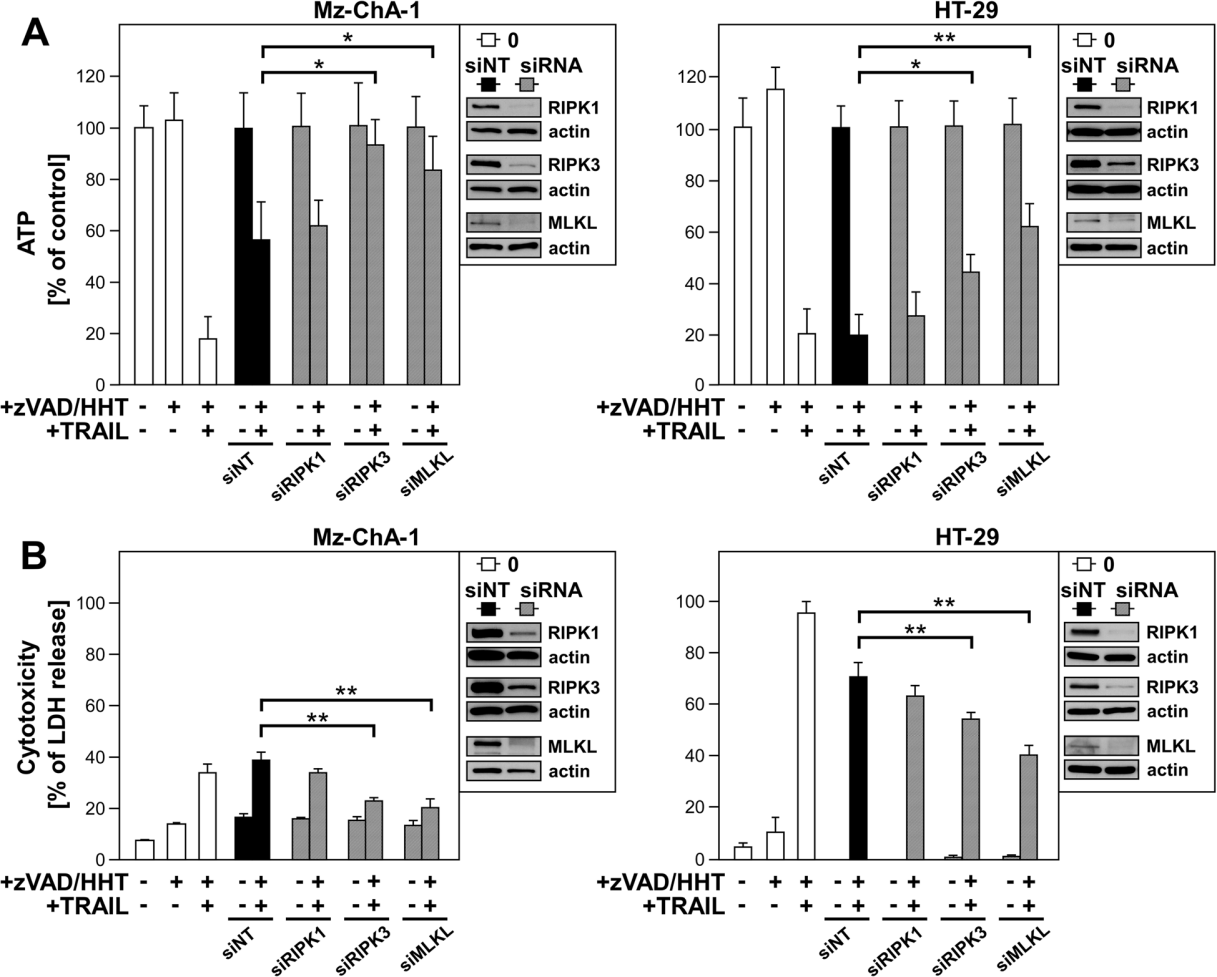
Downregulation of RIPK3 and MLKL protects from TRAIL/zVAD/HHT-mediated necroptosis. Mz-ChA-1 and HT-29 cells were transfected using siPORT™ Amine with siRNAs specific for human RIPK1, RIPK3, or MLKL, or – as a negative control – with a non-target siRNA (NT) that does not elicit an RNAi response. 72 h after transfection, cells were treated with 50 μM zVAD in combination with 0.1 μM HHT (Mz-ChA-1) or 1 μM HHT (HT-29) for one hour before 100 ng/ml TRAIL were added. After 24 h, the decrease of intracellular ATP levels (A) and release of LDH (B) was determined as a marker for cell viability or cytotoxicity, respectively. ATP and LDH levels are shown relative to controls that were not treated with TRAIL/zVAD/HHT. ATP measurements are shown as means of five independent experiments with five parallel determinations each. In each LDH measurement, one out of two representative experiments with five parallel determinations each is shown. Error bars indicate the corresponding SDs. Asterisks indicate statistical significance (t-test; *, *P* < 0.05; **, *P* < 0.001). Control Western blots of untreated transfected cells were performed to verify the downregulation of endogenous human RIPK1, RIPK3 and MLKL. Detection of actin served as loading control.

To additionally validate the above experiments with inhibitors of RIPK1 and MLKL, and to also include functional targeting of RIPK3, we performed siRNA studies to specifically downregulate RIPK1, RIPK3 and MLKL (Figure 6). Furthermore, we employed two additional independent methods to detect cell death in our samples (measurement of ATP levels (Figure. 6A) and the release of lactate dehydrogenase (LDH) (Figure 6B)). Compared to the negative control (non-target siRNA), downregulation of RIPK1 did not change the levels of cell death significantly after TRAIL/zVAD/HHT stimulation. This is in contrast to the above results using Nec-1s, but might be due to remaining amounts of RIPK1 after downregulation that are still sufficient to promote RIPK1-RIPK3 complex formation and subsequently recruitment of MLKL by RIPK3 to execute necroptosis [41]. In contrast, downregulation of RIPK3 and MLKL resulted in a statistically significant protection of the cells after stimulation with TRAIL/zVAD/HHT, confirming their role in HHT-enhanced TRAIL-induced necroptosis.

### Cells undergoing HHT-enhanced TRAIL-induced necroptosis show typical necrotic morphologies

**Figure 7:**
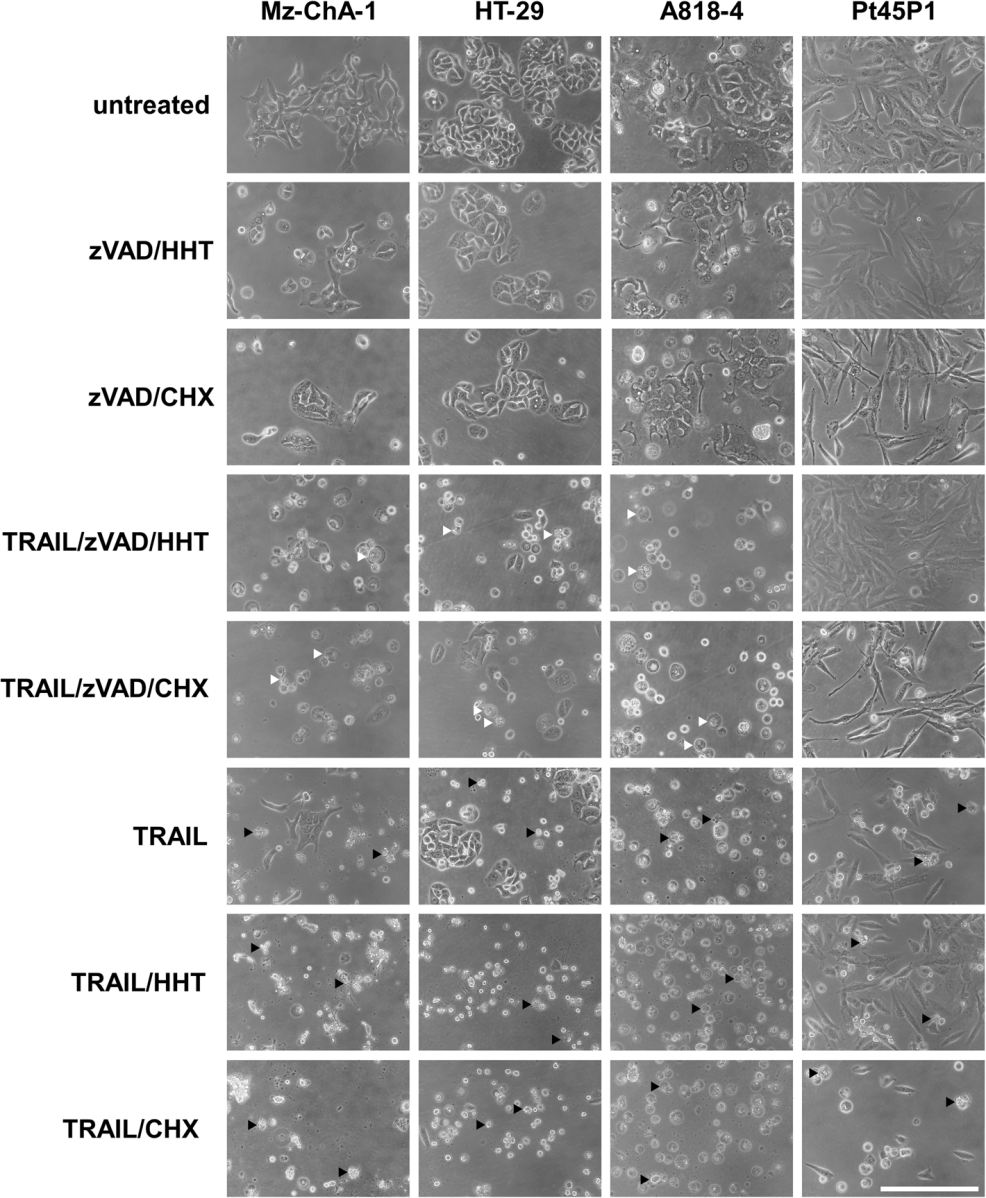
Characteristic morphological features of HHT-enhanced TRAIL-induced necroptosis and apoptosis in tumor cells. Cells were stimulated as indicated in the legend to Figure 2. Micrographs are showing the morphology of cells after 24 h of treatment (to have a direct comparison to the other performed experiments) as indicated on the left. In each micrograph, two typical morphologies of TRAIL-induced necroptosis (white arrows) and apoptosis (black arrows) are indicated. In Pt45P1 cells, no necroptosis was visible as these cells are resistant to this type of cell death. These cells were used as an additional control to monitor if HHT/CHX treatment results in morphological changes of the cells not related to cell death. Scale bar: 100 μm.

In addition to the fundamental differences at the molecular level, apoptosis and necroptosis can be distinguished macroscopically. While in apoptosis the formation of apoptotic vesicles (blebbing) is a characteristic feature, in necroptosis the cells form a balloon-like morphology without nuclear disintegration [42]. In order to clarify whether the addition of HHT caused any atypical alterations of necroptosis that were detectable at the level of cellular morphology, we compared HHT- and CHX-sensitized Mz-ChA-1 and HT-29 cells after induction of necroptosis and apoptosis with TRAIL (Figure 7). In this experiment, we included A818-4 and Pt45P1 cells to clarify if these moderately or not necroptosis-susceptible cell lines would show any morphologic changes upon stimulation or not. In all cases where we induced apoptosis (TRAIL, TRAIL/HHT and TRAIL/CHX treatment), the formation of apoptotic vesicles could be observed (black arrows). These vesicles were not detectable when we induced necroptosis (TRAIL/zVAD/HHT and TRAIL/zVAD/CHX treatment). In these samples, the typical swelling of cells undergoing necroptotic cell death was visible (white arrows). Overall, cells treated with HHT showed no differences in cell morphology compared to cells treated with CHX or without sensitizer. These results implicate that the addition of HHT sensitizes cells for necroptosis (or apoptosis) without altering the morphology or the intracellular signaling pathways of the cell.

### Consecutive induction of necroptosis and apoptosis eliminates cancer cells with higher efficiency

**Figure 8:**
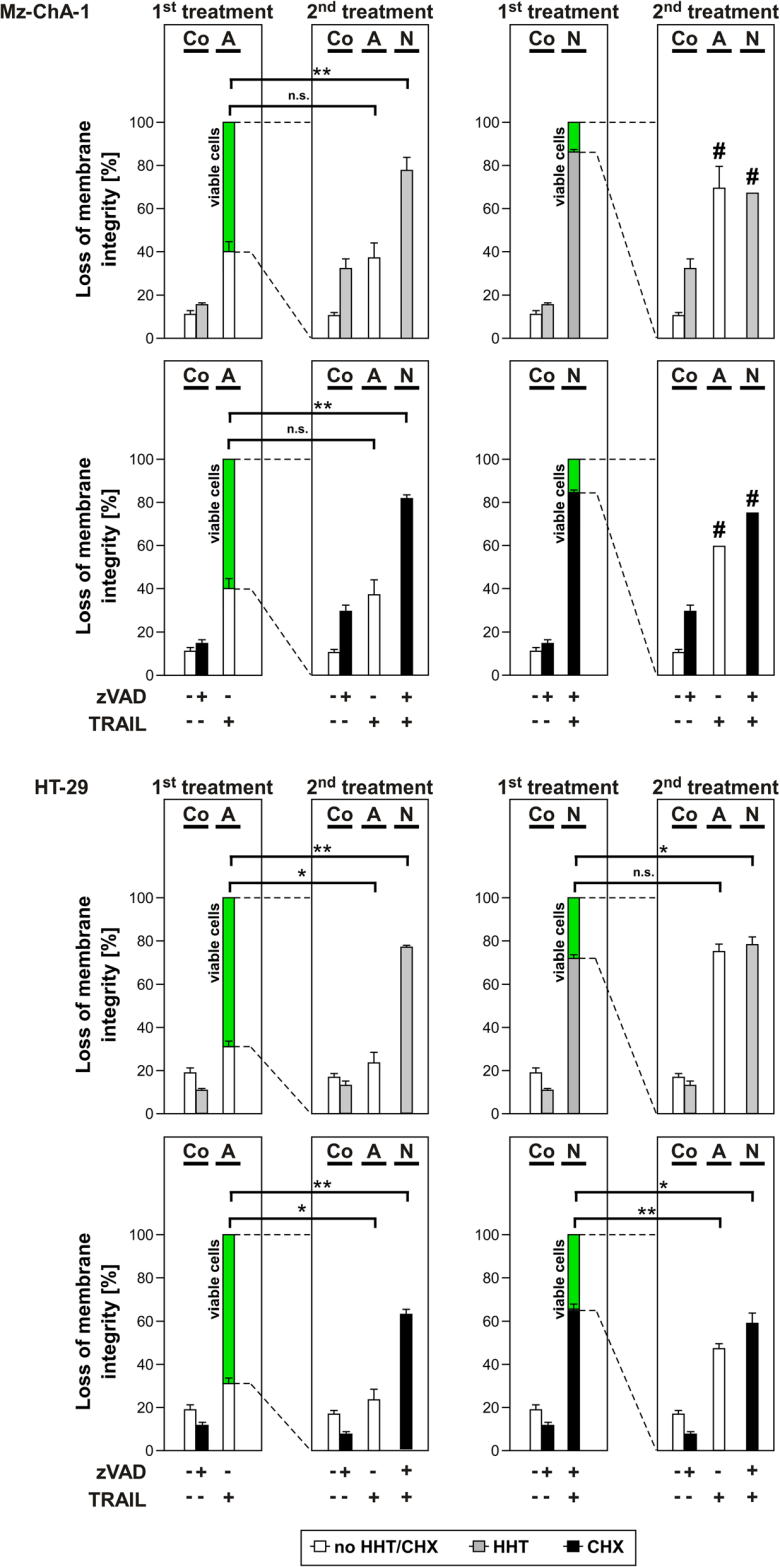
Consecutive induction of necroptosis and apoptosis eliminates cancer cells with higher efficiency. Cells were stimulated as indicated with 50 μM zVAD-fmk or not and with HHT (Mz-ChA-1: 0.1 μM; HT-29: 1 μM) or CHX (Mz-ChA-1: 7.12 μM; HT-29: 17.79 μM). After one hour, 100 ng/ml of TRAIL were added or not as indicated. After 24 h, loss of membrane integrity was measured as a marker for cell death by flow cytometric detection of PI-positive cells (1^st^ treatment). In a parallel experiment, after 24 h of treatment, the supernatant was discarded and adherent, viable cells (green columns) were washed with medium. After 24 h of reconstitution in medium, cells were treated again as indicated. After another 24 h, cells were measured as before via PI-staining (2^nd^ treatment). Each bar represents the means of two independent experiments with three parallel determinations each, error bars indicate the corresponding SDs. In the 1^st^ treatment, both HHT and CHX treatments were performed as part of the same experiment using the same controls. Therefore, the same control values are shown in the two respective panels. The same applies to the 2^nd^ treatment. Asterisks indicate statistical significance (t-test; *, *P* < 0.05; **, *P* < 0.001; n.s., not significant). Due to the high levels of cell death of Mz-ChA-1 cells after the first induction of necroptosis, less than 40,000 cells could be measured after the second induction (#). Co: Control, N: Necroptosis, A: Apoptosis.

Finally, we investigated whether a consecutive induction of apoptosis and necroptosis gives advantages for the elimination of cancer cells (i.e., treatment of cells to induce necroptosis or apoptosis and successive treatment of the surviving cells to induce either apoptosis and necroptosis a second time or alternatively to kill remaining apoptosis-resistant cells by necroptosis and vice versa; Figure 8). In these experiments we could show that after induction of apoptosis (Mz-ChA-1: 40% cell death, HT-29: 31% cell death), a second treatment of the surviving cells with TRAIL to again induce apoptosis resulted in levels of cell death that were comparable to those observed after the initial treatment. In contrast to that, necroptosis as a second stimulus showed significantly higher levels of cell death in the cells that had survived apoptosis (Mz-ChA-1: 78% with HHT and 82% with CHX; HT-29: 77% with HHT and 63% with CHX). This clearly demonstrated that cells surviving apoptosis could be efficiently eliminated via necroptosis, and also confirmed the efficiency of necroptotic cell death. Nevertheless, the induction of necroptosis as a second treatment did not cause higher levels of cell death than in experiments where necroptosis was induced as a first treatment (right panels), indicating that an induction of apoptosis does not further increase the sensitivity of cells for necroptosis. In the reverse approach, cells that survived an initial induction of necroptosis however showed a higher sensitivity towards a consecutive induction of apoptosis (Mz-ChA-1: 70% cell death after HHT and 60% cell death after CHX, HT-29: 75% cell death after HHT and 47% cell death after CHX) compared to a consecutive treatment of solely apoptosis in both treatments (Mz-ChA-1: 40%, HT-29: 31%). In case of a second, consecutive induction of necroptosis in cells that had survived a first induction of necroptosis, we similarly observed high levels of cell death (Mz-ChA-1: 86.5% cell death after HHT and 84.5% cell death after CHX, HT-29: 72% cell death after HHT and 65% cell death after CHX), indicating that the surviving cells had not developed resistance against necroptosis, and possibly pointing to a generally decreased likeliness of cancer cells to become necroptosis-resistant. Especially for Mz-ChA-1 cells, less than 40,000 surviving cells (marked with #) were detectable in flow cytometry after the second treatment. Taken together, these data suggest that a consecutive induction of necroptosis and apoptosis represents a more efficient strategy for the elimination of cancer cells than a single induction of either mode of RCD.

## Discussion

Many tumors are resistant to TRAIL-induced apoptosis and even combinations of TRAIL with sensitizing agents were only partially effective in a number of clinical trials (reviewed in [43]). Thus, alternative ways to kill those resistant cancer cells have to be investigated. The caspase-independent mode of RCD mediated by death receptors termed as necroptosis turned out as a feasible way to eliminate cancer cells in a previous study [7]. However, in most cancer cells, survival pathways counteract the effects of TRAIL-induced RCD, which makes sensitizers such as CHX indispensible to overcome this problem. Unfortunately, those sensitizers are cytotoxic by themselves and cannot be used in treatment of cancer patients.

Here we show that HHT, an U. S. Food and Drug Administration-approved anti-leukemia drug, efficiently sensitizes for TRAIL-induced necroptosis in similar way as known for CHX. We selected cell lines that were highly, moderately, or not susceptible to TRAIL-induced necroptosis when sensitized with CHX and compared this to HHT sensitization. We found that the tested cells reacted with comparable levels of cell death. In agreement with previous data we could show that HHT is also able to sensitize for TRAIL-induced apoptosis [30]. This resulted in higher levels of PARP-1 as well as caspase-3 cleavage, whereas no such cleavage products were detectable in necroptosis. In addition, no cleavage or increased activity of caspase-8 was observed in necroptotic samples compared to untreated cells, whereas cleavage and activation was clearly detectable in apoptotic samples. Taken together, these data clearly confirm that necroptosis and not apoptosis was induced by treatment with TRAIL/zVAD/HHT.

In necroptosis triggered by death receptors, activation of RIPK1 and RIPK3 to form the necrosome is a critical event [44]. Recently, MLKL has also been identified as an additional component that interacts with RIPK3. For TNF-induced necroptosis, MLKL with mutated phosphorylation sites prevented the activation of the necrosome [39,45]. Our Western blot data and the outcome of experiments with the inhibitors Nec-1s and NSA clearly show that HHT-sensitized necroptosis depends on this RIPK1/RIPK3/MLKL signaling that forms the necrosome, in the same manner as CHX-sensitized necroptosis. In addition, our morphological studies show that HHT does not alter the cell morphology within the effective doses for sensitization. This underlines our findings that HHT can substitute CHX to sensitize cancer cells to necroptosis. Importantly, we show that a stepwise treatment with different combinations of necroptosis and apoptosis can result in better elimination of cancer cells. For example, Mz-ChA-1 cells that were more resistant to apoptosis after the first round of treatment could be eliminated via induction of necroptosis in the second round of treatment. Interestingly, these cells could also be sensitized for apoptosis after a first treatment via necroptosis. In addition, we show that cells that survived an initial induction of necroptosis could be further efficiently killed by a second induction of necroptosis. These findings imply a possible application of HHT as a sensitizer in the elimination of cancer cells that are apoptosis resistant and, because HHT is an already U. S. Food and Drug Administration-approved anti-leukemia drug, it may be used in different combinations in patients with apoptosis resistant tumors in future experiments. However, as the next step, HHT-based treatments (consecutive and/or in combination) have to be investigated in mouse xenotransplantation models for their efficiency to kill apoptosis-resistant cancer cells by necroptosis.

As a potent inhibitor of translation, HHT combined with TRAIL may negatively affect healthy cells, as for example reported for the anti-cancer proteasome inhibitor bortezomib [46]. It has been shown that induction of apoptosis by HHT in combination with TRAIL reduced the growth of tumor xenotransplants (HT-29) in immunodeficient mice within the therapeutic window [30]. Recent studies clearly demonstrate the anti-cancer effects of HHT also in patients with acute promyelocytic leukemia (APL). Patients with HHT treatment showed an improved 5-year overall survival rate of 92.6 ± 0.6% (versus 89.9 ± 0.5%; *P* > 0.05) and a 5-year disease free survival rate of 100% (versus 86.8 ± 0.6%; *P* > 0.05) [47]. This and other studies demonstrate that HHT can be safely used in patients, and also suggests that HHT-sensitized necroptosis may likewise safely be employed in future therapy regimens in cancer patients.

As an important point to consider for future therapies, the authors of a recent study [48] raise the intriguing concept that within a tumor, yet unidentified mechanisms of regulated necrosis cause the death of tumor cells within the central core, and in consequence provoke neovascularization, which in turn promotes growth of the residual tumor. In that sense, a tumor would benefit from driving itself into necroptosis rather than being eliminated. However, in this model, only the central core of a tumor is “sacrificed” to necroptosis to promote the accelerated growth of the remaining, neovascularized peripheral part of the tumor. In a future treatment of patients however, necroptosis would be induced in a way to target and eliminate all cells of a tumor within a short timeframe, a) probably leaving not enough time to allow for neovascularization, and b) hopefully not allowing peripheral tumor cells around the central core to survive and benefit from the central cells dying from necroptosis. With regard to the question whether necroptosis is beneficial or detrimental for tumor growth, Colbert and coworkers have recently demonstrated that in pancreatic ductal adenocarcinoma, patients with low tumoral MLKL expression have a poor prognosis [49], indicating that high levels of MLKL expression (and thus susceptibility to necroptosis) are beneficial for patients and detrimental for tumors. Nevertheless, a final resolution of this question will have to await further studies, such as the analysis of orthotopic xenotransplantation models in mice and finally, studies in patients.

## Methods

### Cell lines and culture conditions

The gallbladder adenocarcinoma cell line Mz-ChA-1 and the pancreatic adenocarcinoma cell lines A818-4 and Pt45P1 have been described [50-52]. The colorectal adenocarcinoma cell line HT-29 cells was originally obtained from the American Type Culture Collection. Mz-ChA-1, A818-4 and Pt45P1 cells were cultured in RPMI 1640 (Life Technologies, Darmstadt, Germany) supplemented with 10% v/v FCS, 10 mM glutamine and 1 mM sodium pyruvate. HT-29 cells were cultured in McCoy’s supplemented with 10% v/v FCS, 10 mM glutamine. All cells were kept in a humidified incubator containing 5% w/v CO_2_. Necroptosis was induced by addition of human recombinant TRAIL (SuperKillerTRAIL™, Enzo, Lausen, Germany) in combination with benzyloxycarbonyl-Val-Ala-Asp(OMe)-fluoromethylketone (zVAD-fmk; Bachem, Heidelberg, Germany) and CHX (Sigma-Aldrich, Deisenhofen, Germany) or HHT (Santa Cruz Biotechnology, Heidelberg, Germany). Nec-1s and NSA were purchased from Merck Millipore, Darmstadt, Germany.

### Cytotoxicity assays

Cell death was measured by propidium iodide uptake after loss of membrane integrity. For flow cytometric analysis, cells were seeded onto 12-well plates at 70% confluence. 24 hours after treatment, cells were detached by accutase (Life Technologies), collected and washed once in PBS/5 mM EDTA. Pelleted cells were resuspended in PBS/5 mM EDTA containing 2 *μ*g/ml propidium iodide (PI), and subsequently analyzed in a FACSCalibur flow cytometer (BD Biosciences, San Diego, CA, USA) at red fluorescence.

### Western blot

Whole cell lysates were prepared with TNE lysis buffer (50 mM Tris pH 8.0, 150 mM NaCl, 1% v/v NP-40, 3 mM EDTA, Complete protease inhibitor mixture (Roche, Mannheim, Germany)) on ice. Proteins (20 μg per lane) were separated by SDS-PAGE, transferred onto nitrocellulose membranes and reactive proteins were detected with antibodies against RIPK1 (610459, BD Biosciences), RIPK3 (PAB0287, Abnova, Heidelberg, Germany), MLKL (2103620, Sigma-Aldrich), pMLKL (phospho S358, ab187091, Abcam, Cambridge, UK), PARP-1 (9542, Cell Signaling, Danvers, USA), caspase-8 (1C12, 9746, Cell Signaling), cleaved caspase-3 (9661, Cell Signaling) or actin (A1978, Sigma-Aldrich) by chemiluminescence (LumiGLO, Cell Signaling).

### RNA interference

The siRNAs specific for human RIPK1 (D-004445-03), RIPK3 (D-003534-01) as well as the negative control siRNA (#1, D-001206-13) were obtained from Thermo Scientific (Schwerte, Germany). The siRNA specific for human MLKL (S47087) was purchased from Life Technologies. Mz-ChA-1 and HT-29 cells were transfected with siPORT™ Amine agent (AM4502, Life Technologies). For each transfection (96-well-based assay for ATP and LDH measurements), 0.6 μl of siPORT Amine agent was mixed with 9.4 μl of Opti-MEM® medium (11058021, Life Technologies) tlsb-.19pt?>and incubated at room temperature for 10 min. Afterwards, 2 pmol/μl of siRNA were added, incubated for additional 10 min at room temperature before 80 μl of cell suspension at a density of 6 × 10^3^ cells was added. Cells were incubated for additional 72 h at 37°C before ATP and LDH measurements were performed. For Western blot analysis, the same procedure was performed in 12-well plates using 10 times the volume of each reagent.

### ATP and LDH measurements

Measurement of intracellular ATP was performed with the CellTiterGlow® luminescent cell viability assay (G7570, Promega, Mannheim, Germany) and luminescence intensity was measured on white 96 well flat bottom plates with an Infinite M200 microplate reader (Tecan, Crailsheim, Germany). The release of LDH was detected directly in cell culture by the cytotoxicity detection KitPlus (04744934001, Roche) according to the manufacturer’s protocol and measured with an Infinite M200 microplate reader (absorbance at 450 nm, background absorbance at the reference wavelength of 600 nm). The average absorbance values of the samples and controls, each in 5 repeats, were calculated by subtraction of the background absorbance from the measured absorbance. As controls, the spontaneous LDH activity in cell culture medium (low control, Lc) and the maximum releasable LDH activity after cell lysis (high control, Hc) were determined. The cytotoxicity (% of LDH release) was calculated as: [(treated mean – Lc mean)/(Hc mean – Lc mean)] × 100.

### Analysis of caspase-8 activity

Activity of caspase-8 was measured as described previously [4]. Mz-ChA-1 and HT-29 cells were collected and lysed in a buffer containing 10 mM Hepes pH 7.4, 142 mM KCl, 5 mM MgCl2, 1 mM EGTA, 0.2% v/v NP40, 1 mM DTT and 2 mM Pefabloc (Roche). Positive controls were produced out of 20 μg of cell lysate, that was equilibrated for 1 h at 30°C after the addition of 1 mM dATP and 10 μM cytochrome c to permit activation of caspases. Subsequently, 100 μl of caspase buffer (20 mM Pipes, 100 mM NaCl, 10 mM DTT, 1 mM EDTA, 0.1% w/v CHAPS, 10% w/v sucrose, pH 7.2) containing 100 μM zIETD-afc (benzyloxycarbonyl-Ile-Glu(OMe)-Thr-DL-Asp(OMe)-7-aminotrifluoromethylcoumarin, Merck Millipore) were added to 10 μl of cytosolic extract (0.5 μg protein) and incubated at 37°C. The release of afc was then measured as emission at 505 nm upon excitation at 405 nm using an Infinite M200 fluorimeter equipped with a thermostated plate reader.

### Microscopy

For documentation of cell morphology, images from unfixed cells were obtained using an Axiovert 10 microscope (Zeiss, Oberkochen, Germany) and a DS-5 M-L1 digital sight camera system (Nikon, Düsseldorf, Germany).

### Statistical analysis

*P* values were calculated using Student’s t-test. Statistical significance is denoted by *P < 0.05 and **P < 0.001.

## Abbreviations

CHX: Cycloheximide
HHT: Homoharringtonine
LDH: Lactate dehydrogenase
PI: Propidium iodide
RCD: Regulated cell death
SD: Standard deviation
TRAIL: Tumor necrosis factor-related apoptosis-inducing ligand
zIETD-afc: Benzyloxycarbonyl-Ile-Glu(OMe)-Thr-DL-Asp(OMe)-7-aminotrifluoromethylcoumarin
zVAD-fmk: Benzyloxycarbonyl-Val-Ala-Asp(OMe)-fluoromethylketone
